# Supplementary material for: Ankyrin-B is lipid-modified by S-palmitoylation to promote dendritic membrane scaffolding of voltage-gated sodium channel NaV1.2 in neurons
Source: Front Physiol. 2023 Mar 30;14:959660. doi: 10.3389/fphys.2023.959660 (PMC10098127; doi:10.3389/fphys.2023.959660)
Supplement: Supplementary file 3 [file DataSheet1.pdf]

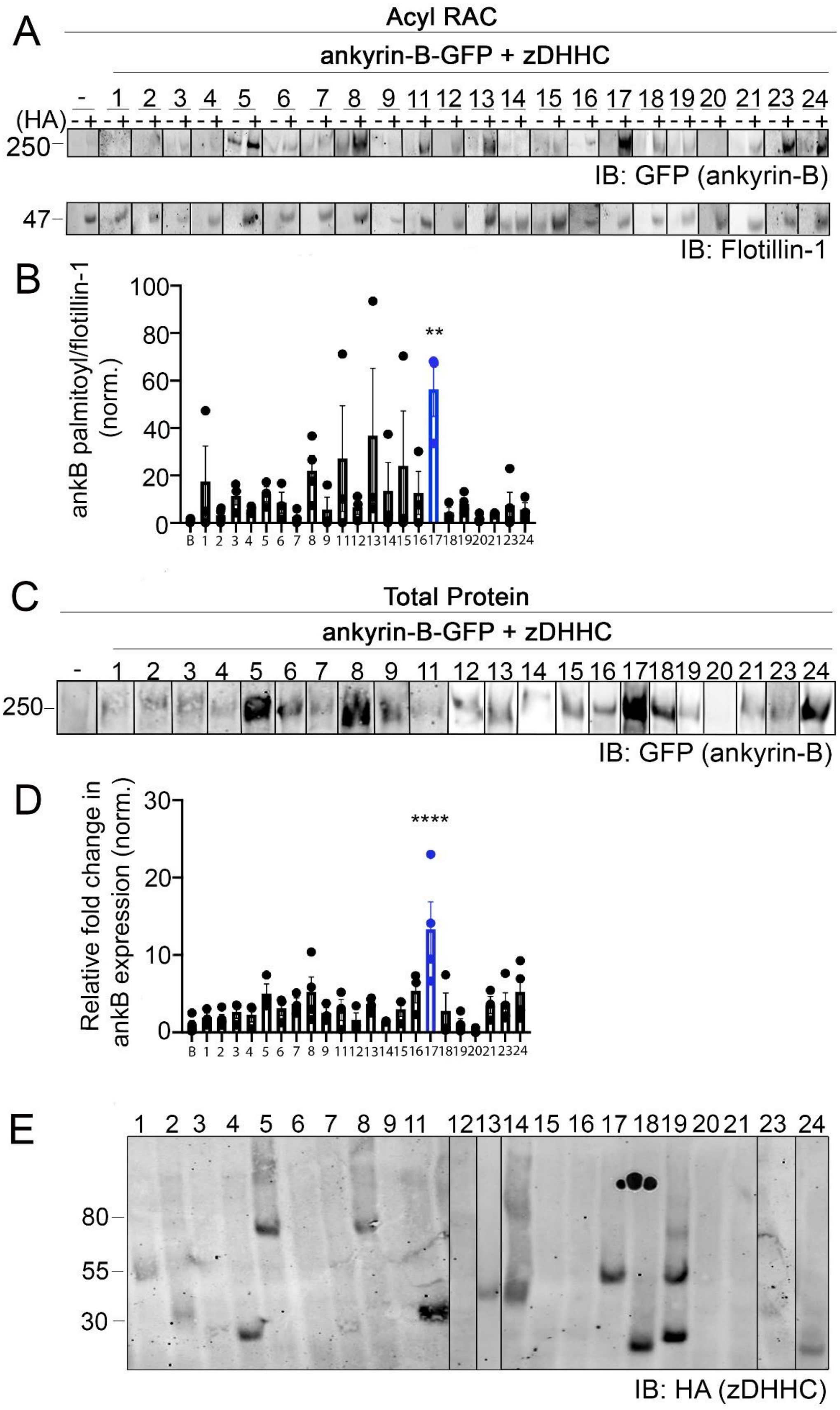
A
Acyl RAC
ankyrin-B-GFP + zDHHC
- 1 2 3 4 5 6 7 8 9 11 12 13 14 15 16 17 18 19 20 21 23 24
(HA)
250
IB: GFP (ankyrin-B)
47
IB: Flotillin-1
B
ankB palmitoyl/flotillin-1 (norm.)
**
C
Total Protein
ankyrin-B-GFP + zDHHC
250
IB: GFP (ankyrin-B)
D
Relative fold change in ankB expression (norm.)
****
E
80
55
30
IB: HA (zDHHC)

Figure S1. zDHHC17 increases ankyrin-B palmitoylation and protein expression in HEK293T cells.

A. Representative western blot showing ankyrin-B-GFP palmitoylation in the absence or presence of each individual zDHHC enzyme. Lysates from HEK cells transiently transfected with 220-kDa ankyrin-B-GFP alone (indicated as "-" in figure) or with each individual zDHHC enzyme were subjected to the Acyl-RAC assay to detect which zDHHC enzymes selectively enhance the palmitoylation of ankyrin-B-GFP compared to ankyrin-B alone. *S*-palmitoylation of ankyrin-B-GFP is detected using an antibody against GFP, as shown by the anti-GFP signal in '*+HA'* lane, compared to background signal in the negative control -*HA* lane. **B**. Quantification of *A*. zDHHC17 significantly enhances *S*-palmitoylation of ankyrin-B-GFP by approximately 91 fold compared to ankyrin-B-GFP alone (N=3). For each condition, the palmitoylation signal is calculated by subtracting the '*-HA'* lane signal from that of the '*+HA'* lane and normalizing to the flotillin signal from the '*+HA'* lane, and further normalizing to the average of ankyrin-B alone signal to get the relative fold change in ankyrin-B-GFP palmitoylation. Significance ($p = 0.0012$) was determined using an ordinary one-way ANOVA and Tukey's post-hoc multiple comparisons test. **C.** Representative western blot showing ankyrin-B-GFP total protein expression in the absence or presence of each individual zDHHC enzyme. **D**. Quantification of *C*. zDHHC17 significantly increases the protein expression of ankyrin-B-GFP by approximately 13 fold compared to ankyrin-B alone, while none of the other zDHHC enzymes significantly increase ankyrin-B-GFP expression (n=3), ****p<0.0001 (one-way ANOVA, Tukey's post-hoc). **E.** Representative western blot showing expression of the HA-tagged zDHHC enzymes expressed in the samples from panels A and C. Approx. molecular weight for each western blot indicated in kDa.

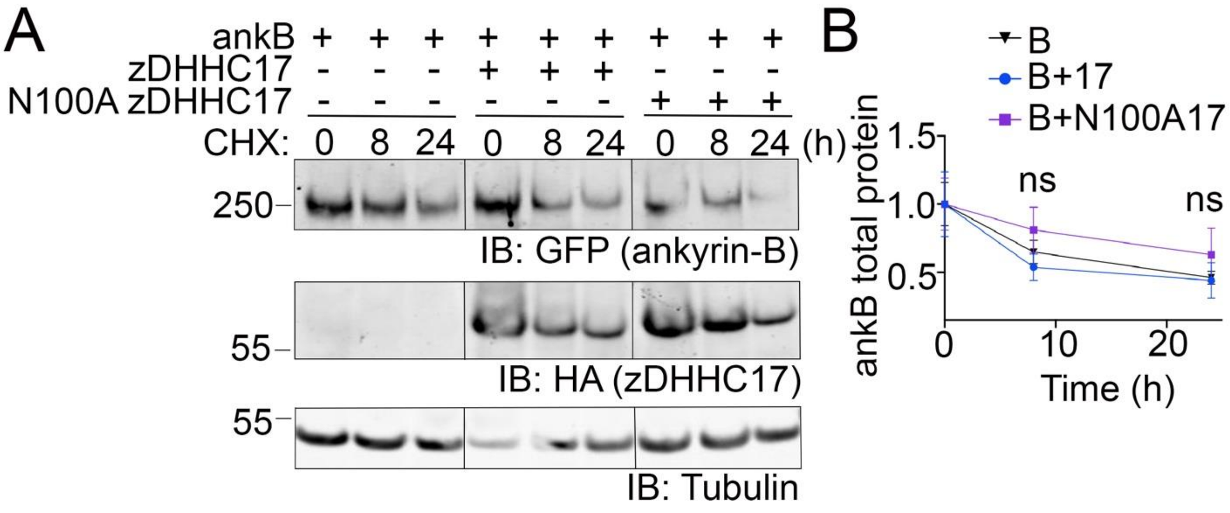

Figure S2. zDHHC17 does not alter protein stability of ankyrin-B in heterologous cells by the cycloheximide (CHX) chase assay.

A. Representative western blot showing ankyrin-B protein expression alone or in the presence of WT zDHHC17 or N100A zDHHC17 after 8 and 24 hours of cycloheximide treatment. HEK293T cells transiently transfected with 220-kDa ankyrin-B-GFP alone or with WT zDHHC17 or N100A zDHHC17 were treated with 100 µg/mL CHX and lysates were collected after 8 hours and 24 hours of CHX treatment before western blotting. Ankyrin-B expression is detected using an antibody against GFP. Absence or presence of zDHHC17 is detected using an antibody against HA. Tubulin is used as a loading control. For gel shown, all samples were run on the same gel to account for difference in baseline protein level; black line delineates spliced portion of the gel containing a condition not relevant to this figure. **B.** Quantification of *A*. Co-expression of zDHHC17 or N100A zDHHC17 does not alter the stability of ankyrin-B, compared to ankyrin-B in the absence of zDHHC17. For each condition, each time point is normalized to tubulin, and further normalized to Time 0h. Results are from N=6 for each condition; not significant (ns) by multiple unpaired *t-test*.

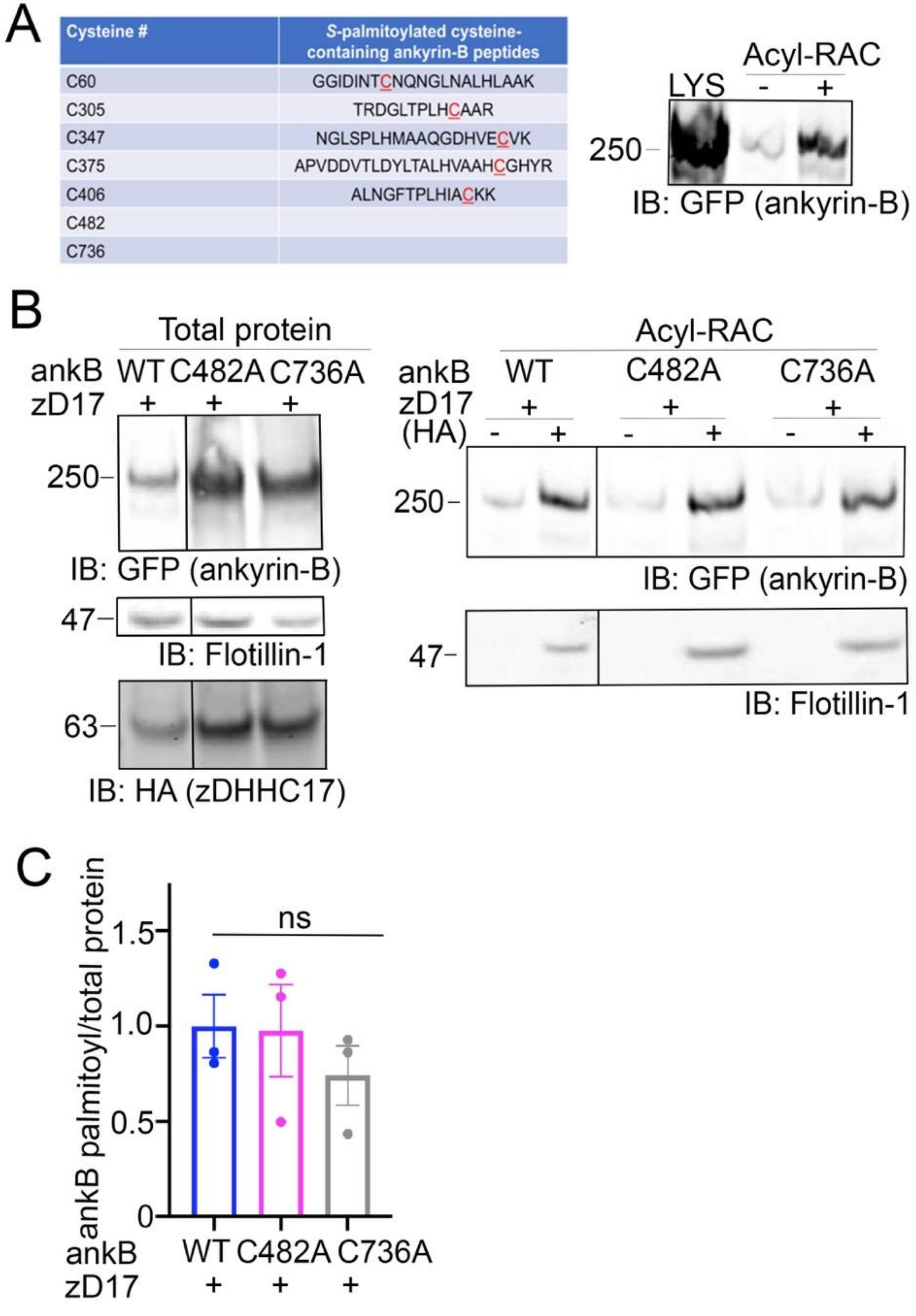

Figure S3. Cys482 and Cys736 in ankyrin-B are not *S*-palmitoylated.

A. Five cysteine-containing peptides were identified by mass spectrometry from HEK293T cells co-transfected with ankyrin-B-GFP and zDHHC17 processed for the Acyl-RAC assay. These cysteine-containing peptides corresponded to Cys60, Cys305, Cys347, Cys375, and Cys406 in ankyrin-B. Western blot demonstrates efficiency of the Acyl-RAC assay for sample containing overexpressed ankyrin-B-GFP and zDHHC17-HA submitted for mass spectrometry analysis. **B.** Validation that the two cysteines, Cys482 and Cys736 not identified as palmitoylated in the mass spectrometry analysis were indeed not palmitoylated by Acyl-RAC. Western blot shows total protein levels of ankyrin-B-GFP (*left*) and palmitoylation levels of ankyrin-B-GFP (*right*) from lysates of HEK293T cells transiently co-transfected with WT ankyrin-B-GFP, C482A ankyrin-B-GFP, or C736A ankyrin-B-GFP and zDHHC17 processed for the Acyl-RAC assay. No change in the palmitoylation levels of C482A or C736A ankyrin-B-GFP were observed compared to WT ankyrin-B-GFP, as evidenced by the unchanged level of GFP signal in the '+HA' lane for these two mutants compared to WT ankyrin-B-GFP. All conditions shown are run on the same blot; black line delineates spliced portion of the gel containing a condition not relevant to this figure. **C.** Quantified ankyrin-B-GFP *S*-palmitoylation levels normalized to total ankyrin-B protein levels for each condition, relative to palmitoylation levels of WT ankyrin-B-GFP co-expressed with zDHHC17. Data from N=3 independent replicates per condition from *B*. ns; one-way ANOVA, Tukey's post-hoc test.

Movie 1

tdTomato-tagged Syt1 particle (red) movement in axons of DIV4 *Ank2*$^{flox/flox}$ mouse hippocampal neurons transfected with Syt1-tdTomato. Time-lapse images were acquired at a rate of 5 frame/s using a confocal microscope and are displayed at a rate of 10 frames/s. Time stamp indicates seconds. Particle tracking analysis output from KymoToolBox shown side-by-side for comparison (red/retrograde, blue/stationary, and green/anterograde particles on greyscale image).

Movie 2

tdTomato-tagged Syt1 particle (red) movement in axons of DIV4 *Ank2*$^{flox/flox}$ mouse hippocampal neurons transfected with Syt1-tdTomato plus Cre-2A-BFP. Time-lapse images were acquired at a rate of 5 frame/s using a confocal microscope and are displayed at a rate of 10 frames/s. Time stamp indicates seconds. Particle tracking analysis output from KymoToolBox shown side-by-side for comparison (red/retrograde, blue/stationary, and green/anterograde particles on greyscale image).

Movie 3

tdTomato-tagged Syt1 particle (red) movement in axons of DIV4 *Ank2*$^{flox/flox}$ mouse hippocampal neurons transfected with Syt1-tdTomato plus Cre-2A-BFP and ankyrin-B-GFP. Time-lapse images were acquired at a rate of 5 frame/s using a confocal microscope and are displayed at a rate of 10 frames/s. Time stamp indicates seconds. Particle tracking analysis output from KymoToolBox shown side-by-side for comparison (red/retrograde, blue/stationary, and green/anterograde particles on greyscale image).

Movie 4

tdTomato-tagged Syt1 particle (red) movement in axons of DIV4 *Ank2*$^{flox/flox}$ mouse hippocampal neurons transfected with Syt1-tdTomato plus Cre-2A-BFP and AAAAA-ankyrin-B-GFP. Time-lapse images were acquired at a rate of 5 frame/s using a confocal microscope and are displayed at a rate of 10 frames/s. Time stamp indicates seconds. Particle tracking analysis output from KymoToolBox shown side-by-side for comparison (red/retrograde, blue/stationary, and green/anterograde particles on greyscale image).

Movie 5

Ankyrin-B-GFP (green) movement in axons of DIV4 *Ank2*$^{flox/flox}$ mouse hippocampal neurons transfected with Syt1-tdTomato plus Cre-2A-BFP and ankyrin-B-GFP. Time-lapse images were acquired at a rate of 5 frame/s using a confocal microscope and are displayed at a rate of 10 frames/s. Time stamp indicates seconds. Particle tracking analysis output from KymoToolBox shown side-by-side for comparison (red/retrograde, blue/stationary, and green/anterograde particles on greyscale image).

Movie 6

AAAAA-Ankyrin-B-GFP (green) movement in axons of DIV4 *Ank2*$^{flox/flox}$ mouse hippocampal neurons transfected with Syt1-tdTomato plus Cre-2A-BFP and AAAAA-ankyrin-B-GFP. Time-lapse images were acquired at a rate of 5 frame/s using a confocal microscope and are displayed at a rate of 10 frames/s. Time stamp indicates seconds. Particle tracking analysis output from KymoToolBox shown side-by-side for comparison (red/retrograde, blue/stationary, and green/anterograde particles on greyscale image).
